# Antioxidant and Anti-Inflammatory Activities of Safflower (*Carthamus tinctorius* L.) Honey Extract

**DOI:** 10.3390/foods9081039

**Published:** 2020-08-02

**Authors:** Li-Ping Sun, Feng-Feng Shi, Wen-Wen Zhang, Zhi-Hao Zhang, Kai Wang

**Affiliations:** 1 Institute of Apicultural Research, Chinese Academy of Agricultural Sciences, Beijing 100093, China; sunliping01@caas.cn (L.-P.S.); shi9914@126.com (F.-F.S.); zhangwen_w@126.com (W.-W.Z.); talent-zzh@163.com (Z.-H.Z.); 2 College of Animal Science (College of Bee Science), Fujian Agriculture and Forestry University, Fuzhou 350002, China

**Keywords:** safflower (*Carthamus tinctorius* L.) honey, chemical analysis, anti-inflammatory, antioxidant, NF-κB, Nrf-2

## Abstract

Safflower honey is a unique type of monofloral honey collected from the nectar of *Carthamus tinctorius* L. in the *Apis mellifera* colonies of northwestern China. Scant information is available regarding its chemical composition and biological activities. Here, for the first time, we investigated this honey’s chemical composition and evaluated its in vitro antioxidant and anti-inflammatory activities. Basic physicochemical parameters of the safflower honey samples in comparison to established quality standards suggested that safflower honeys presented a good level of quality. The in vitro antioxidant tests showed that extract from *Carthamus tinctorius* L. honey (ECH) effectively scavenged DPPH and ABTS^+^ free radicals. In lipopolysaccharides (LPS) activated murine macrophages inflammatory model, ECH treatment to the cells inhibited the release of nitric oxide and down-regulated the expressions of inflammatory-relating genes (iNOS, IL-1β, TNF-α and MCP-1). The expressions of the antioxidant genes TXNRD, HO-1, and NQO-1, were significantly boosted in a concentration-dependent manner. ECH decreased the phosphorylation of IκBα and inhibited the nuclear entry of the NF-κB-p65 protein, in LPS-stimulated Raw 264.7 cells, accompany with the increased expressions of Nrf-2 and HO-1, suggesting that ECH achieved the anti-inflammatory effects by inhibiting NF-κB signal transduction and boosting the antioxidant system via activating Nrf-2/HO-1 signaling. These results, taken together, indicated that safflower honey has great potential into developing as a high-quality agriproduct.

## 1. Introduction

Honey used widely as a food and medicine [1]. It is often studied due to its biological activities, among them the anti-inflammatory, anti-bacterial, and anti-oxidation properties are the most published [2,3,4,5]. Others include the resistance to bromobenzene-induced liver injury in mice [6] and potential treatment for colitis [7]. However, there are some special honeys, which come from particular floral sources, for example, Manuka, which can play an anti-ulcer role through antioxidant and anti-inflammatory effects [4,8], or Gelam honey, which was able to effectively inhibit airway inflammation in an ovalbumin-induced allergic asthma mouse model [9]. There are significant differences in the antioxidant activities among different honeys [10]. Safflower (*Carthamus tinctorius* L.) honey is brewed by honeybees collecting safflower nectar, which is a local speciality honey type of Xinjiang Providence, China. This honey is mainly produced in the Balruk Mountains of Xinjiang (82°12′–83°30′ longitude in the East and 45°24′–46°3′ latitude in the north), and contains a variety of natural sweeteners with bioactive components [11,12,13], which is the largest safflower planting base in China. Our recent preliminary results suggested that the safflower honey has several superiorities, such as comprehensive and rich nutrients, and a large number of bioactive substances [14]. Nevertheless, detailed information is still unavailable regarding its chemical composition and biological activities, which limits the application and development of this special agriproducts.

Recent decades, several studies reported on the anti-inflammatory and antioxidant activities of honey, which are mainly attribute to its abundant phenolic and flavonoid contents [15,16]. Honey contains rich phenolic acids and flavonoids, which contribute to the antioxidant, antimicrobial, anti-inflammatory, anti-proliferation, anti-cancer, and anti-metastasis effects [17]. Studies showed that these compounds in honey were able to inhibit the pro-inflammatory activity of nitric oxide synthase (iNOS) and had anti-inflammatory effects [17]. Bangladesh honey samples are rich in phenolic acids and flavonoids with high antioxidant potential [18]. Honeys from specific floral sources or collected by different bee species, given their special and rare compounds, such as certain phenolic acids and flavonoids, for instance, in stingless honey, can show anti-inflammatory and antioxidant effects [19]. Data published with Camellia honey presented an antioxidant effect closely related to its phenolic content [20]. We therefore inferred that the safflower honey also some activities, such as the anti-inflammatory and antioxidant properties. In our previous studies, we evaluated the anti-inflammatory activities by the bee products using the bacterial lipopolysaccharides (LPS) challenged murine macrophage model (RAW 264.7 cells), in which model a number of typical inflammatory responses can be mimicked in vitro, including the releases of inflammatory mediators, accompanying with the oxidative stress to the cells. LPS induced RAW 264.7 cells were shown to be with an increase in nitric oxide (NO) release [21,22], which is mediated via the activation of the inflammation related-nuclear factor kappa-B (NF-κB) signaling pathway [23], and thereafter induced oxidative stress. NO has a significant correlation with oxidative stress [24], and its release depends on the expression of inducible NO synthase (iNOS). LPS-activated Raw 267.4 cells also lead to the rapid phosphorylation and degradation of IκBα [25]. The phosphorylation of IκB-α or IκB-β can activate the NF-κB signaling pathway and promote NF-κB-p65 protein in the nucleus. However, the NF-κB-p65 protein can mediate the synthesis/releases of tumor necrosis factor alpha (TNF-α), interleukin-1β (IL-1β), monocyte chemoattractant protein 1 (MCP-1), which further regulate the transcription of other inflammatory mediators [26,27,28]. In addition, the NF-E2-related factor 2 (Nrf-2) signaling activation regulates the expression of a series of downstream antioxidant factors (4-nitroquinoline-N-oxide (NQO), HO-1 as well as the thioredoxin reductase(TXNRD)) [29,30]. Activation of these internal anti-oxidant enzymes alleviated the cells against oxidative stress [31].

In the current study, *Carthamus tinctorius* L. safflower honey were collected to carry out a screening of physical and chemical indicators and the extract from *Carthamus tinctorius* L. safflower honey (ECH) were obtained for preliminary characterizations on major main phenolic acids and flavonoid components based on the high-performance liquid chromatography–quadrupole-time of flight mass spectrometry (HPLC-QTOF-MS). Furthermore, we tested the in vitro free radical scavenging activities by ECH and its anti-inflammatory and antioxidant potentials were evaluated in in LPS-activated Raw 264.7 murine macrophages.

## 2. Materials and Methods

### 2.1. Chemicals and Reagents

Primary antibodies including IκB-α, p-IκBα, and NF-κB-p65 were purchased from Cell Signaling Technology (Danvers, MA, USA). Nrf-2, HO-1, and *β*-actin were purchased from Cambridge Bio (Boston, MA, USA). The NanoDrop 2000 Ultra Micro Spectrophotometer was purchased from Thermo Fisher Scientific (Pittsburgh, PA, USA). Gallic acid, sodium nitrite, the Prime Script TM RT Master Mix kit and TB Green^®^ Premix Ex Taq TM were purchased from Biotech Co. Ltd. (Shanghai, China). Protein loading buffer, 20% SDS, electroporation solution and Tris-HCl buffer were purchased from Solarbio (Beijing, China). BCIP/NBT alkaline phosphatase developer and 40% Acr-Bis were purchased from Beyotime Biotechnology (Shanghai, China). The CCK-8 kit was purchased from Dojindo Laboratories (Kumamoto, Japan). Amberlite XAD-2 resin was purchased from Sigma-Aldrich Trading Co. Ltd. (Shanghai, China). Other chemicals, including the LPS (*Escherichia coli* 0127: B8) and alkaline phosphatase-conjugated secondary antibody (anti-rabbit IgG) and the standards applied in the chemical analysis were purchased from Sigma (St. Louis, MO, USA).

### 2.2. Safflower Honey Samples and Physical Deteriminations

Safflower honey samples were obtained from three *Apis mellifera* L. colonies during the safflower (*C. tinctorius* L.) flower season in 2018. Each honey from the single hive was considered as one sample, 500 g each. The apiaries were located in the Yumin County, Tacheng City, China, which were belongs to bases of Jiangsu Rigao Bee Products Co., Ltd. (Xuyi, China). We collected the capped comb honeys in the day, and transfer them to the refrigerator at 4 °C, afterwards the honey samples were taken to the lab and storage at −20 °C until usage. The moisture, free acid, amylase, hydroxymethylfurfural, fructose, glucose, sucrose, and ash contents of the Xinjiang safflower honey samples were determined by the method specified in CODEX STAN 12-1981. Melissopalynology was applied to analyze the botanical origin of the pollens in the safflower honeys, following pervious published method [32]. The typical pollen grain in a safflower honey sample was shown in the Figure S1.

### 2.3. Extraction on the Safflower Honey

The preparation of the extract from *C. tinctorius* safflower honey (ECH) refers to the method of Mu et al. [33] and was improved. XAD-2 resin was soaked in 95% ethanol for 24 h, washed two to three times with ultrapure water until there was no ethanol, and then placed for standby. We weighed 800 g of the Xinjiang safflower honey sample, mixed it with hydrochloric acid solution (adjusted using hydrochloric acid pH = 2 with ultrapure water) at 1:5 (*w*/*v*), and used an ultrasonic instrument for 30 min until it dissolved. The activated XAD-2 resin was weighed to 800 g, treated with ultrasonic to make it free of air, then mixed with Xinjiang safflower honey aqueous solution, mixed evenly for 1 h, and left standing overnight. We discarded the supernatant, added XAD-2 resin into the glass column, washed with 2 volumes of hydrochloric acid water (pH = 2) and 3 volumes of ultra-pure water, and then eluted with 8 volumes of ethanol to collect the eluate, evaporated the ethanolic extract to a solid residue with a rotary evaporator with vacuum, and then dissolved the residue in 15 mL of pure water. This aqueous solution was extracted with 20 mL of ethyl acetate. We collected the ethyl acetate layer, repeated this four times, for each extraction for 20 min. The collected ethyl acetate layer was blown dry with nitrogen to obtain the resulting ECH, and placed in a −20 °C refrigerator for storage. We dissolved the ECH with an appropriate amount of ethanol for the further usages.

### 2.4. Preliminary Analysis of ECH Phenolic Flavonoids by HPLC-QTOF-MS

The HPLC-QTOF-MS analysis method of ECH was established by our laboratory [34]. Chromatographic conditions involved using a proshell 120 EC-C18 column (100 × 2.1 mm, particle size 2.7 µm), typical parameters of chromatographic were as follows: column temperature, 30 °C; injection volume, 5 µL; flow rate, 0.2 mL/min. The elution procedure is shown in Table 1.

Mass spectrometry conditions were conducted with an electrospray (ESI) ion source in negative ion mode The typical parameters of the mass spectrometer were as follows: drying gas temperature, 350 °C; drying gas flow rate, 6 L/min; sprayer pressure, 35 psi; capillary voltage 3500 V; atomizing gas temperature, 350 °C; and atomizing gas flow rate, 9 L/min. Qualitative and quantitative analysis were carried out by accuracy mass and extracted ion chromatography (EIC). Polyphenolic compounds ion chromatograms were extracted by Mass Hunter Qualitative Analysis software (Agilent Technologies) for ECH.

### 2.5. In Vitro Free Radical Scavenging Ability Determination Experiment

#### 2.5.1. DPPH Free Radical Scavenging Experiment

For the determination of the DPPH· clearance rate, we referred to WU et al.’s method [35], and made certain adjustments. We placed 100 µL each of DPPH working solution and ECH into a 1.5 mL centrifuge tube, shook and mixed, and react at room temperature in the dark for 30 min. We took 100 µL of the reaction liquid to a 96-well plate (100 µL/well), and measured the absorbance at 517 nm. For A1, the same method was used to determine the absorbance when adding 100 µL of 95% ethanol solution instead of DPPH working solution. The absorbance of the blank group (100 µL DPPH solution and 100 µL of 95% ethanol solution) was recorded as A0. The calculation formula of the clearance rate is:

$$\mathrm{clearance}\;\mathrm{rate}\%=1-\frac{A1-A2}{A0}\times 100$$

The removal ability of the sample is expressed by IC_50_.

#### 2.5.2. ABTS^+^ Free Radical Scavenging Experiment

For the determination of the ABTS^+^ clearance rate, we referred to YANG et al. [36] and made certain adjustments. The ABTS solution was generated by the reaction of 15 mL 7 mM ATBS solution and 246 µL 140 mM potassium persulfate aqueous solution in the dark for 16 h. When used, it was diluted with methanol to the absorbance of 0.70 ± 0.02 at 734 nm. We placed 250 µL ABTS methanol working solution and 150 µL ECH (to dissolve) in a 1.5 mL centrifuge tube, shook and mixed, avoided light for the reaction for 10 min. We took 150 µL of the reaction liquid to a 96-well plate (150 µL/well), and measured the absorbance value, recorded as A1. The same method was used to determine the absorbance when adding 250 µL of methanol instead of ABTS methanol working solution. The absorbance of the blank group (250 µL ABTS solution and 150 µL (ECH solvent) solution) was recorded as A0, with parallel values. The calculation formula of the clearance rate is:

$$\mathrm{clearance}\;\mathrm{rate}\%=1-\frac{A1-A2}{A0}\times 100$$

The removal ability of the sample is expressed by IC_50_.

### 2.6. Cell Experiment

Raw 264.7 cells were a gift from Professor Hu Fuliang, college of Animal Science, Zhejiang University. Raw 264.7 cells were cultured with DMEM high glucose medium containing 10% heat-inactivated fetal bovine serum and double antibodies (streptomycin (100 µg/mL) and penicillin (100 IU/mL)) at 37 °C Incubation was in a 5% CO_2_ incubator [37], to maintain the stable growth of the cells.

#### 2.6.1. ECH Measurement on Cell Relative Survival Rate

Raw 264.7 cells in the logarithmic growth phase were inoculated into 96-well plates with 1 × 10^5^ cells/mL, 100 µL per well, incubated overnight in a 5% CO_2_ incubator at 37 °C, until the cell adherence reached 70–80%. The cells were incubated with 2.5 µg/mL, 5 µg/mL, 10 µg/mL, 15 µg/mL, or 20 µg/mL ECH for 24 h, and the culture medium was aspirated. The cells were washed with new cell culture medium for two to three times, and a blank control group was set. We added 10 µL CCK-8 reagent and cell culture medium into each well, incubated for 3 h, and then the absorbance at 450 nm was determined [38,39].

#### 2.6.2. Determination of the Nitric Oxide (NO) Concentration in LPS-Induced Cells Treatment with ECH

The NO concentration was measured with a Griess reagent. We referred to the method of Lee et al. and adjusted [40]. Raw 264.7 cells in the logarithmic growth phase were inoculated into 24-well plates with 1 × 10^5^ cells/mL, 500 µL per well, incubated in a 37 °C, 5% CO_2_ incubator, until the cell adherence reached 70–80%, and we set the DXMS positive control group, blank control group, LPS treatment control group, and experimental group. To the experimental group, we successively added 2.5 µg/mL and 5 µg/mL ECH for 1 h, followed by LPS (1 µg/mL) for 24 h. The cell culture fluid was collected, centrifuged at 5000 r/min for 10 min, and the supernatant was collected. We mixed 100 µL of each sample with an equal volume of Griess reagent and added these samples to a 96-well plate, and incubated at room temperature for 10 min. We measured the absorbance at 540 nm.

#### 2.6.3. ECH Detection of the mRNA Expression Related to Inflammation and Oxidation in LPS-Induced Cells

Raw 264.7 cells in the logarithmic growth phase were inoculated into 24-well plates with 1 × 10^5^ cells/mL, 500 µL per well, incubated in a 37 °C, 5% CO_2_ incubator, until the cell adherence reached 70–80%, and we set the DXMS positive control group, blank control group, LPS treatment control group, and experimental group. To the experimental group, we successively added 2.5 µg/mL and 5 µg/mL ECH and incubated for 1 h, and then induced with LPS (1 µg/mL) for 6 h. The cells were collected, and then the total cell RNA was extracted using a CarryHelix RNA extraction kit, and its concentration and purity were determined with a NanoDrop 2000 ultramicro spectrophotometer. Using 1 µg of extracted total RNA as a template, the PrimeScript^®^ TM RT Master Mix reverse transcription kit was used to perform reverse transcription of the cDNA, and the product was placed in a −20 °C refrigerator for use. Real-time quantitative PCR was performed with a TB Green^®^ Premix Ex TaqTM kit. The total volume of the reaction system was 10 µL: TB Green^®^ Premix Ex TaqTM 5.0 µL, cDNA template 0.2 µL, RNase Free dH_2_O 4.4 µL, upstream and downstream primers 0.2 µL each, related to use. The primer sequences are shown in Table 2.

#### 2.6.4. ECH Detection of Related Protein Expression in LPS-Induced Cells

Raw 264.7 cells in the logarithmic growth phase were inoculated in 6-well plates with 1 × 10^5^ cells/mL, 1 mL per well, and incubated in a 37 °C, 5% CO_2_ incubator, so that the cell adherence reached 90%, and we set the DXMS positive control group, blank control group, LPS treatment control group, and experimental group. To the experimental group we successively added 2.5 µg/mL and 5 µg/mL ECH and incubated for 1 h, and then induced with LPS (1 µg/mL) for 0.5 h. The collected cells were washed twice with PBS, and inhibited with *NP-40* protein lysate Cellular protein was extracted from the reagent, and the concentration of the extracted protein was measured by BCA. The protein samples of each group of cells were detected by immunoblotting. The total sample loading was 20 µg of total protein, with β-actin as a reference, through 12% SDS-PAGE gel electrophoresis, transfer, hybridization, alkaline phosphatase color development and other steps to obtain the hybridization bands of IκBα, P-IκBα, Nrf-2 and HO-1 [42].

#### 2.6.5. Effect of ECH on LPS-Induced Nuclear Localization of p65 (NF-κB)

Raw 264.7 cells in the logarithmic growth phase were inoculated into the slide confocal small dish with 1 × 10^5^ cells/mL, incubated in a 37 °C, 5% CO_2_ incubator overnight, and we set up the DXMS positive control group, blank control group, LPS treatment control group, and experimental group. To the experimental group, we added 5 µg/mL of ECH and incubated for 1 h. Then, we induced with LPS (1 µg/mL) for 0.5 h. A methanol-acetone mixture (1:1, *v*/*v*) was used as the fixing solution for 30 min. Permeabilization was performed with 0.5% Triton X-100 for 30 min, blocked with 10% serum blocking solution for 30 min at room temperature. We added primary antibody (NF-κB-p65) (1:50 dilution) and secondary antibody goat anti-rabbit (IgG) (1:500 dilution) and incubated for 1 h, with DAPI (4′,6-diamidino-2-phenylindole) (1:2000 dilution) stained nuclear processing coverslips, observed with a laser confocal scanning microscope, and analyzed the results [42].

### 2.7. Statistics Analysis

All experimental data were obtained from at least three repeated experiments. Data are presented as the Mean ± SD, and the statistical significance between two groups was determined using Student’s *t*-test, and a *p* value of <0.05 was considered as statistically significant.

## 3. Results

### 3.1. Physical and Chemical Analysis on the Safflower Honey

All data regarding the physical and chemical indicators of Xinjiang safflower honey (see Table 3), using the relevant methods in the EU standard, revealed better results than the standard values cited. Among them, the moisture content was 18.2%, which meets the EU standard for honey (not more than 20%). The acidity (1 mol/L sodium hydroxide titration) was 25.0 mL/kg, which is lower than the EU standard limit of 50 mL/kg. Hydroxymethylfurfural, sucrose, and ash were not detected; the three were far below the relevant EU standard limits. The fructose content was 36.9%, the glucose content was 25.2%, and the total of the two was 62.1%, higher than the EU standard (not less than 60%); the amylase value was 21.1 mL/(g·h), which is much higher than the EU standard (not less than 8), which is twice the EU requirements.

### 3.2. Preliminary Analysis of Phenolic Flavonoids in ECH by HPLC-QTOF-MS

HPLC-QTOF-MS detection of ECH was carried out with 24 phenolic flavonoid standards. As shown in Table 4, eight phenolic acids were detected, with a total content of 7.197 mg/kg honey. Vanillic acid and p-hydroxybenzoic acid were the highest phenolic acids, with 3.196 mg/kg honey and 1.524 mg/kg honey, respectively. Protocatechualdehyde was not detected. Seven flavonoids were detected, with a total content of 7.633 mg/kg honey, with quercetin and myricetin being the highest, with 3.196 mg/kg honey and 1.524 mg/kg honey, respectively. Rutin, quercetin-3-O-glucoside, kaempferol, naringenin and pinobanksin were low in content. Morin, luteolin, diosmetin, pinocembrin, galanin, caffeic acid phenethyl ester, chrysin and kaempferol-3-O-glucoside were not detected. As far as the content of safflower honey phenolic acid flavonoids is concerned, the phenolic acid components were characterized by vanillic acid and p-hydroxybenzoic acid, and the flavonoids were characterized by quercetin and myricetin. The diversity and content of phenolic acid and flavonoids play a positive role in the antioxidant and anti-inflammatory activities of safflower honey.

### 3.3. In Vitro Antioxidant Free Radical Scavenging Capacity of ECH

DPPH and ABTS^+^ free radical scavenging experiments are commonly used to evaluate natural antioxidants [15]. It can be seen from Table 5 that the concentration of ECH inhibiting 50% DPPH free radical was 68.23 ± 0.40 µg/mL, and the concentration of ECH inhibiting 50% ABTS^+^ free radical was 81.88 ± 0.54 µg/mL.

### 3.4. In Vitro Antioxidant, Anti-Inflammatory Activies by ECH

#### 3.4.1. Effects of ECH on Raw 264.7 Cell Survival

**Figure 1:**
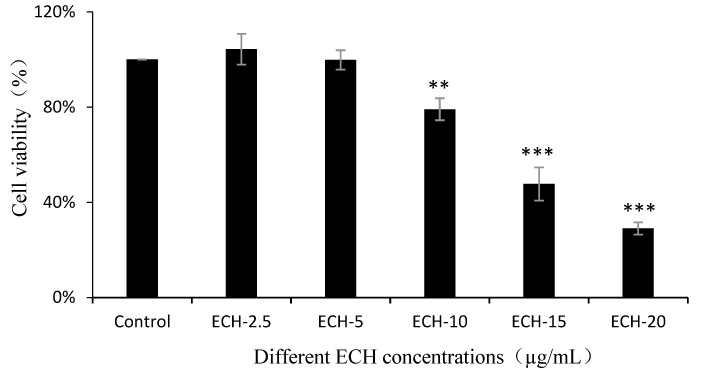
The effects of various concentrations of ECH on RAW 264.7 cell viability. Raw 264.7 cells were pretreated with ECH (2.5 to 20 µg/mL) or not for 24 h, cell viability was tested using the CCK-8 method. ** (*p* < 0.01) and *** (*p* < 0.001) indicates significant difference compared with the control group.

By treating Raw 264.7 cells with ECH in concentrations from 2.5 to 20 µg/mL and using the CCK-8 reagent to detect cell viability, we found the most suitable ECH concentration to ensure that adding ECH concentration to Raw 264.7 cells produced no obvious toxic effects (see Figure 1). Compared with the control group, there was no significant difference in the growth of Raw 264.7 cells when the ECH concentration were 2.5 µg/mL and 5 µg/mL. When the ECH concentration was 10 µg/mL, the Raw 264.7 cells growth was highly significantly inhibited. When the ECH concentrations were 15 µg/mL and 20 µg/mL, the inhibitory effect on the growth of Raw 264.7 cells was highly significant. Therefore, the safe concentration of ECH available in this experiment was 2.5–5 µg/mL.

#### 3.4.2. Effect of ECH on LPS-Induced Nitric Oxidase (NO) Release in RAW 264.7 Cells

**Figure 2:**
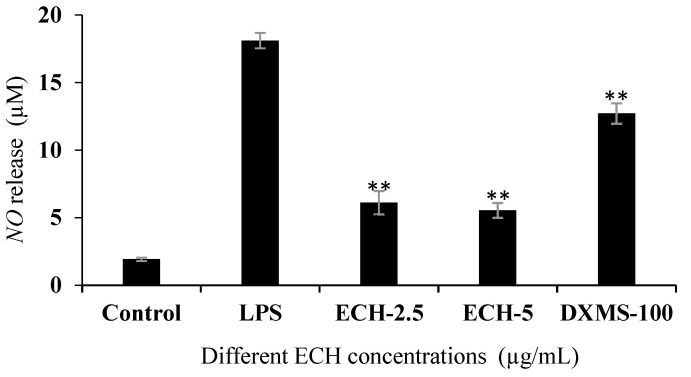
The effects of ECH on the nitric oxide (NO) release in LPS-activated macrophages. RAW 264.7 cells were pretreated with/without indicated concentrations of ECH or dexamethasone (100 µg/mL, positive control) for 1 h then stimulated with LPS (1 µg/mL) for 24 h. NO concentrations in the cell culture medium were measured using the Griess method. ** (*p* < 0.01) indicates significant difference compared with the LPS group.

A typical Griess method was used to determine the amount of NO released by different treatment cells (see Figure 2). Dexamethasone (DXMS) treatment at 100 µg/mL (referred as DXMS-100) to the LPS-activated cells was used as the positive control group. Compared with the Control group, the concentrations of NO releases in Raw 264.7 cells was increased about nine times following LPS treatment, indicating the model of Raw 264.7 cells inflammation model was successfully established. After co-treatment with ECH and LPS, the NO release decreased by about two-thirds compared with the LPS group, and the decreasing effects even better than the DXMS-100 group. Therefore, ECH was shown to effectively reduce the amount of NO released during macrophage inflammation induced by LPS.

#### 3.4.3. Effect of ECH on LPS-Induced Inflammation and Oxidation-Related Gene Expression in RAW 264.7 Cells

**Figure 3:**
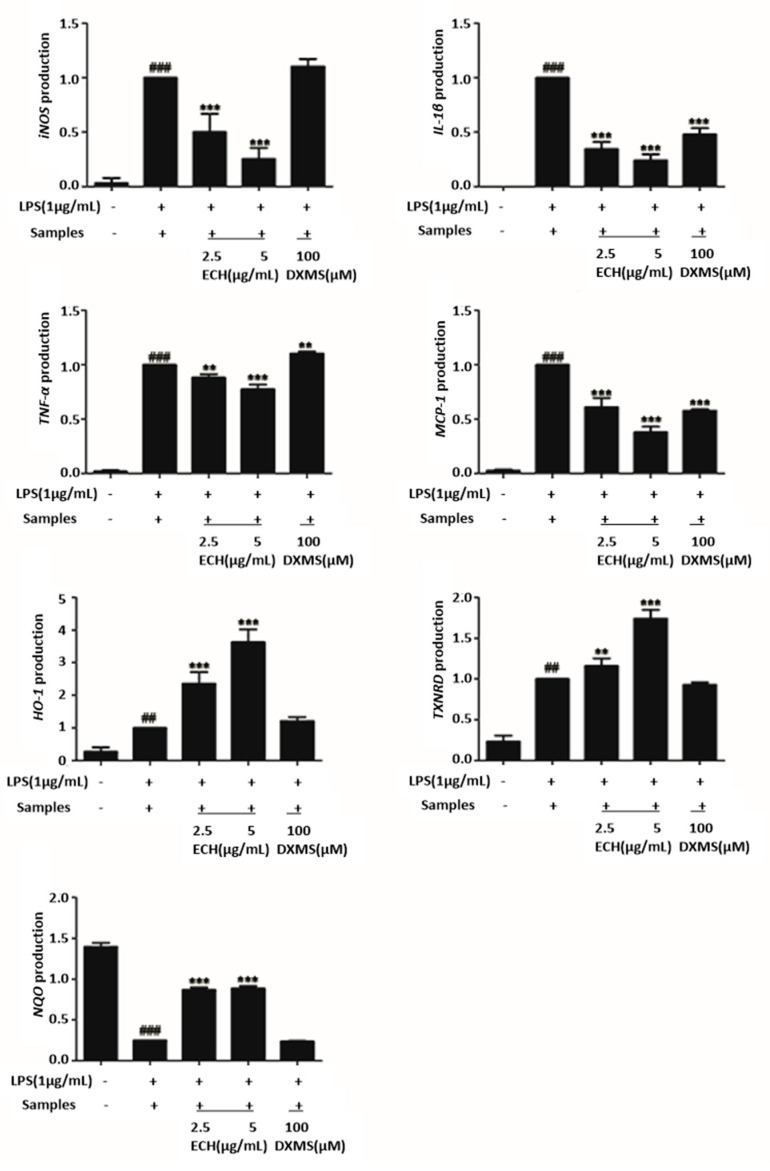
Effects of ECH on the expressions of antioxidant and inflammatory genes in LPS stimulated RAW 264.7 cells. RAW 264.7 cells were pretreated with/without indicated concentrations of ECH or dexamethasone (100 µg/mL, positive control) for 1 h then stimulated with LPS (1 µg/mL) for 6 h. mRNA expression in RAW 264.7 cells were measured using RT-qPCR. ** (*p* < 0.01) and *** (*p* < 0.001) indicates significant difference compared with the LPS group. ## (*p* < 0.01) and ### (*p* < 0.001), compared with the normal control group.

Next, RT-qPCR was applied to detect the expression levels of inflammation and oxidation-related genes in LPS-induced Raw 264.7 cells (see Figure 3). After LPS treatment, the expression of NQO decreased significantly, the expression of iNOS, IL-1β, TNF-α and MCP-1 increased significantly. Compared with cells treated with LPS alone, the expressions of iNOS, IL-1β, TNF-α and MCP-1 in ECH-treated groups were decreased, and the inhibitory effect was stronger with the increase of the ECH concentration. For HO-1, TXNRD, and NQO, ECH significantly increased these antioxidant-related gens expressions.

#### 3.4.4. Effect of ECH on the Expressions of Inflammation and Anti-Oxidant Signaling Related Proteins in LPS-Activated RAW 264.7 Cells

**Figure 4:**
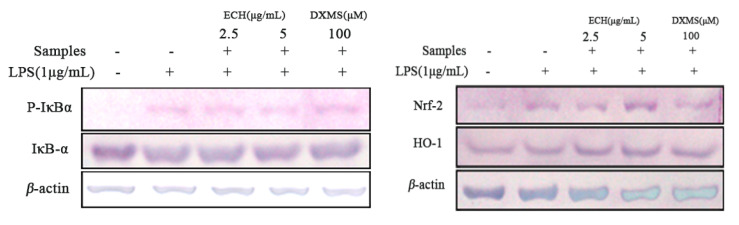
Effect of ECH on the expressions of inflammation and anti-oxidant signaling related proteins in LPS-induced activated RAW 264.7 cells RAW 264.7 cells were pretreated or not with indicated concentrations of ECH for 1 h then were activated with LPS (1 µg/mL) for 30 min (left) or 6 h (right). Whole cell lysates were analyzed by Western blotting analysis using specific antibodies.

The expression of inflammatory and anti-oxidation-related proteins in LPS-induced Raw 264.7 cells was detected by western blot (Figure 4). The expression level of P-IκBα in the LPS group was significantly higher than that of IκBα, however, after ECH treatment, the expression of P-IκBα in the LPS induced group was significantly reduced compared to the LPS treatment group only. IκBα phosphorylation was inhibited, and 5 µg/mL was found with a more potent inhibitive effects thant the low dosage group (2.5 µg/mL). The expression levels of Nrf-2 and HO-1 increased significantly in cells induced by LPS after ECH pretreatment. When the concentration of ECH increased, the expression levels of Nrf-2 and HO-1 increased more obviously.

#### 3.4.5. Effect of ECH on the Nuclear Localization of NF-κB-p65 Induced by LPS

**Figure 5:**
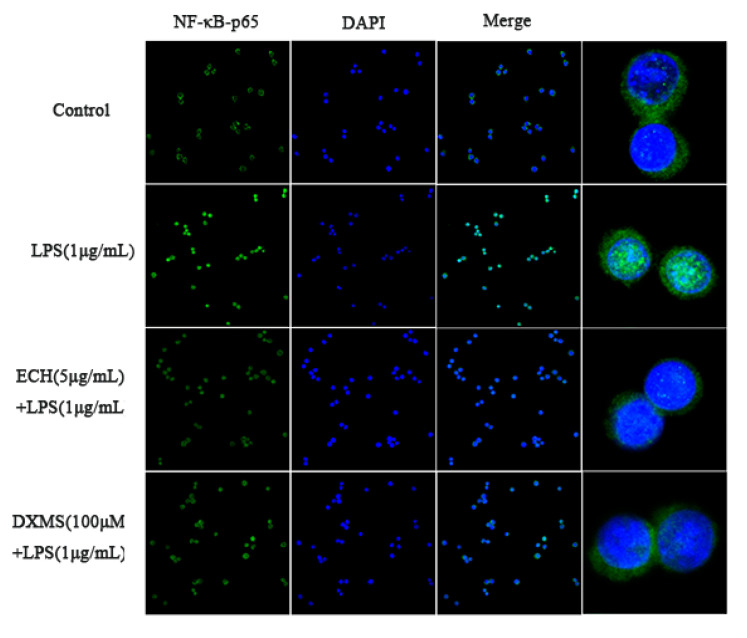
The inhibited effects of ECH on the transport from the cytoplasm to the nucleus of NF-κB-p65 proteins.

A laser confocal scanning microscope was used explore the effect of 5 µg/mL ECH on the nuclear localization of NF-κB-p65 in LPS-induced inflammatory cells (see Figure 5). Compared with the control group, NF-κB-p65 entry into the nucleus was significantly increased during LPS treatment alone, indicating with the activation of NF-κB. After 5 µg/mL ECH incubation and LPS treatment, NF-κB-p65 entry into the nucleus was significantly inhibited; DXMS-100 group treatment of NF-κB-p65 into the nucleus was also significantly inhibited.

## 4. Discussion

Using the methods recommended in the European Community Directives, we found that all the physical and chemical indexes results of Xinjiang safflower honey samples were in line with the requirements. Hydroxymethylfurfural is a substance produced by honey during storage or heating. It is an important criterion for evaluating the quality of honey [43,44]. As the honey storage time becomes longer or the heating temperature rises higher, the more its content increase. Amylase is an important indicator of freshness in honey [1]. Honey enzymes will inactivate (or the value will drop) when honey is heated to a temperature higher than 40 °C. The amylase value measured in our experiment was 21.1 ± 0.3 mL/g honey, which is much higher than the 8 mL/g honey of EU standard (2001/110/EC). We did not detect hydroxymethylfurfural nor sucrose in the safflower honey samples. The moisture complied with EU standard 2001/110/EC to not be higher than 20 g/100 g. The safflower honey results for moisture were better than carob honey from Morocco as studied by Redouan et al. [45], The carob honey moisture was 19.5 g/100 g. Therefore, the Xinjiang safflower honey samples were fresh and mature honey.

As polyphenols endowed honey with distinct color, taste, flavor and biological activities. Polyphenol analysis is therefore considered as an important tool for determining the quality of honey. A total number of 14 polyphenolic compounds were preliminary characterized using the HPLC-Q-TOF/MS analysis, and the sum of each component content was 14.83 mg/kg honey. Solid phase extraction (SPE) using the Amberlite XAD-2 resin is one of the most widely used extraction methods for isolating individual phenolic and flavonoid compounds in honey [46]. In the present study, we didn’t measure the phenolic compounds recovery, which is a major limitation. Nevertheless, the honey was reported with a mean recovery of 43.7% (ranging from 6.7%, gallic acid, to 65.2%, naringin) [47]. In addition, the discrepancies of the extraction recoveries have been found among different types of honey, which might be attributed to sugars as well as some other matrix constitutions. Among them, eight phenolic acid components, and the vanillic acid and p-hydroxybenzoic acid contents were higher, which were 3.196 mg/kg honey and 1.524 mg/kg honey, respectively, accounting for 65.6% of the total phenolic acid content. Protocatechuic acid, gallic acid, caffeic acid, ferulic acid, and other active components were also detected. In addition, seven flavonoid components were detected, among which quercetin and myricetin were higher, at 5.342 mg/kg honey and 1.021 mg/kg honey, respectively, accounting for 83.4% of the total flavonoid content. Rutin, quercetin-3-O-glucoside, kaempferol and naringenin were lower. Protocatechualdehyde, kaempferol-3-O-glucoside, morin, luteolin, diosmetin, chrysin, pinocembrin, galanin, and CAPE, were not detected. We propose the first reason is that different honey plants, different regions, and other environments have a great impact on the polyphenol composition. Secondly, our polyphenol extraction method has a low extraction rate of flavonoids. In a previous study, Bangladesh honey samples (Mustard flower honey, Kalijira honey, Padma flower honey, and Teel/sesame honey) had higher levels of caffeic acid and benzoic acid in phenolic acid substances, while gallic acid, kaempferol, myricetin, and naringenin were also detected [18]. Sundarban, Bangladesh, which is a multifloral honey collected from the largest mangrove forest in the world, showed various phenolic compounds, including gallic acid, vanillic acid, pyrogallol, trans-cinnamic acid, and other phenolic substances, as well as naringin, rutin, and quercetin flavonoids. Among all these phenolic compounds, trans-cinnamic acid and naringin had the highest contents [48]. There are more than 30 kinds of polyphenols in honey. Naringenin and hesperetin typically only appear in specific honeys, such as orange honey [17].

The DPPH· and ABTS^+^ free radical scavenging capacity experiments used the IC_50_ (semi-inhibitory rate, i.e., the concentration of the analyte required to scavenge 50% of free radicals) as the criterion for evaluating the antioxidant activity. The literature showed that due to the rich polyphenol content, honey has a very significant antioxidant effect. Five types of honey in Croatia showed high DPPH· free radical scavenging activity (black locust honey 125.48 mg/mL, lime honey 42.77 mg/mL, sage honey 25.04 mg/mL, chestnut honey 16.02 mg/mL, and honeydew honey 8.69 mg/mL [49]), In eight kinds of carob honey from different areas of Morocco, the DPPH free radical IC_50_ values ranged from 12.54 mg/mL to 23.52 mg/mL [45] and seven kinds of Turkish single flower honey (Heather, Oak, Chestnut, Pine, Astragalus, Acacia, Lavender) demonstrated, ABTS^+^·free radical IC_50_ values from 0.06 g/mL–3.68 g/mL [50]. In our experiment, the DPPH and ABTS^+^·free radical IC_50_ values of ECH were 68.23 ± 0.40 µg/mL and 81.88 ± 0.54 µg/mL, respectively, and they had high antioxidant activity.

In this experiment, ECH can significantly reduce the mRNA levels of the related inflammatory factors iNOS, IL-1β, TNF-α and MCP-1, while up-regulating the expression of the antioxidant genes HO-1, TXNRD and NQO. Due to the expression of iNOS when suppressed, the amount of NO released is reduced accordingly. Through the detection of IκBα phosphorylation and the NF-κB signaling pathway, we found that ECH was likely to inhibit the phosphorylation of IκBα to produce P-IκBα, thereby inhibiting the activation of the NF-κB signaling pathway and reducing inflammation. The Nrf-2/HO-1 signaling pathway demonstrated a protective effect against oxidative stress. Nrf-2 can regulate the gene expression of HO-1 at the transcription level, while HO-1 can inhibit the production of inflammatory factors in LPS-stimulated Raw 264.7 cells and protect the cells under oxidative stress [27]. We measured the expression of Nrf-2 and HO-1, which increaseds in line with the improvement of the ECH concentration. The 2.5 µg/mL and 5 µg/mL ECH anti-inflammatory and anti-oxidant effects were superior to the positive control group Dexamethasone, indicating that ECH has good anti-inflammatory and anti-oxidant effects.

ECH is rich in phenolic acids and flavonoids and also has good anti-oxidation and anti-inflammatory effects. Thus, we speculated that the existence of these compounds provides a material basis for the antioxidant and anti-inflammatory activities of safflower honey extract. Vanillic acid can play an anti-inflammatory and analgesic role through anti-oxidation and inhibiting the production of pro-inflammatory cytokines associated with NF-κB [51]. Protocatechuic acid and p-hydroxybenzoic acid showed significant antioxidant activity in thyme honey [52]. The O_3_-H_15_ bond of gallic acid is easy to break to scavenge free radicals, providing antioxidant effects [53]. Gallic acid can also block LPS-induced activation of TLR4/NF-κB (Toll-like receptor 4/nuclear factor-κB) to inhibit the inflammation of Raw 264.7 cells [54]. Caffeic acid reduces LPS-induced neuroinflammation through the regulation of cytokine networks, down-regulates NF-κB-dependent pro-inflammatory genes, and reduces oxidative stress [55]. Ferulic acid can exert anti-inflammatory activity by inhibiting nitroso-oxidative stress and pro-inflammatory cytokine production [56]. We found that the greatest common denominator of these substances is that they all contain different amounts of hydroxyl groups; thus, we infer that the vast majority of the substances involved in antioxidant activities are all related to these hydroxyl groups. It was reported that quercetin can scavenge active oxygen, inhibit damage caused by oxidative stress, and prevent the development of TNF-α and the secretion of iNOS and IL-1β in Raw 264.7 cells induced by lipopolysaccharide (LPS) [57]. Myricetin exerted anti-inflammatory effects by inhibiting NF-κB-p65 in the NF-κB pathway [58]. Rutin [59] and naringenin [60] alleviate oxidative stress and exert anti-inflammatory effects by regulating mechanisms related to the Nrf-2/HO-1 signaling pathway. Quercetin-3-O-glucoside can inhibit the expression of TNF-α, IL-1β and IL-6 and the activation of NF-κB, and simultaneously up-regulate the expression of Nrf-2 and HO-1, and inhibit the inflammation and oxidation to resist the induction of cisplatin of acute kidney injury in mice [61]. Kaempferol can up-regulate the expression of iNOS in rat articular chondrocytes stimulated by IL-1β, and can inhibit the degradation of IκBα and the activation of NF-κB in rat articular chondrocytes stimulated by IL-1β [62]. Based on this, we propose that these polyphenolic compounds in ECH are the main contributors to the anti-oxidation and anti-inflammatory effects of safflower honey.

In the present study, we must explain the limitations of our experiment. First, as the identified polyphenols might had multiple isoforms and exist in the honey in a very complex form, their quantifications in our honey samples might have some bias. Second, phenolic compounds recovery from XAD-2 resin could to be estimated which can partly counteract the strong matrix effects in the honey. Third, in this study we only preliminary detected several polyphenolic compounds in our safflower honey samples, we still did not propose a “marker” compound in our honey samples, which is with great importance for the standardization and quality control on this special honey type. Nevertheless, our research group are keeping working on the isolation and identification on the marker compound of the safflower honey, which is helpful to discriminate from other commercial honey types. In such work accurate recovery rate will be examined on the single maker compound.

## 5. Conclusions

In summary, we found that the main physical and chemical indicators of safflower monofloral honey samples, a local specialty honey type of Xinjiang Providence, China were in-line with the requirements of EU or Codex standards. We also preliminary characterized 14 polyphenolic compounds in the extracts from *Carthamus tinctorius* L. honey (ECH). ECH showed a strong ability to scavenge DPPH·and ABTS^+^·free radicals in vitro. ECH also inhibited the phosphorylation of *IκBα* to activate the *NF-κB* signaling pathway, promoted the expression of antioxidant genes, thereby inhibiting inflammatory process induced by LPS. Our research provides basic information into development on this special honey in the future.

## Figures and Tables

**Table 1:** Mobile phase elution procedures.

Time (min)	Phase A% (0.1% Formic Acid)	Phase B% (100% Acetonitrile)
0	90	10
0–15	75	25
15–20	70	30
20–30	65	35
30–35	30	70
35–40	30	70
40–42	90	10
42–50	90	10

**Table 2:** The real-time PCR-related primer sequences [25,34,41].

Gene	Upstream Primer Sequence	Downstream Primer Sequence
iNOS	5′-TTTCCAGAAGCAGAATGTGACC-3′	5′-AACACCACTTTCACCAAGACTC-3′
IL-1β	5′-CCAACAAGTGATATTCTCCATGAG-3′	5′-ACTCTGCAGACTCAAACTCCA-3′
TNF-α	5′-CCACGCTCTTCTGTCTACTG-3′	5′-ACTTGGTGGTTTGCTACGAC-3′
MCP-1	5′-AAGAAGCTGTAGTTTTTGTCACCA-3′	5′-TGAAGACCTTAGGGCAGATGC-3′
HO-1	5′-ACATTGAGCTGTTTGAGGAG-3′	5′-TACATGGCATAAATTCCCACTG-3′
TXNRD	5′-AGGATTTCTGGCTGGTATCG-3′	5′-CTCGCTGTTTGTGGATTGAG-3′
NQO	5′-TTCAACCCCATCATTTCC-3′	5′-TCAGGCGTCCTTCCTTATA-3′

**Table 3:** The physical and chemical indicators of safflower honey.

Physical and Chemical Indicators	Result	Standard Limited	Standard Method
Moisture (%)	18.2 ± 0.25	≤20	2001/110/EC
Acidity (mL/kg)	25.0 ± 0.43	≤50	2001/110/EC
Amylase value (mL/(g·h))	21.1 ± 0.36	≥8	2001/110/EC
Hydroxymethylfurfural (mg/kg)	ND	≤40	2001/110/EC
Fructose (%)	36.9 ± 0.26	≥60	2001/110/EC
Glucose (%)	25.2 ± 0.14		
Sucrose (%)	ND	≤5	2001/110/EC
Ash (%)	ND	≤0.1	2001/110/EC

Note: ND means not detected.

**Table 4:** High-performance liquid chromatography combined with a quadrupole time-of-flight mass. spectrometry (HPLC-QTOF/MS) analysis of phenolic flavonoids in safflower.

Compound Name	Standard Curve Equation	R^2^	Concentration Ranges (ng/mL)	RT (min)	[M − H]^−^	Content (mg/kg)
gallic acid	y = 573.42x − 32178.21	0.9983	50–2000	1.765	168.7	0.636
Protocatechuic acid	y = 925.79x + 37631.28	0.9938	50–2000	2.501	153	0.115
protocatechualdehyde	y = 338.47x + 18654.55	0.9991	50–1000	3.589	136.9	ND
p-hydroxybenzoic acid	y = 437.73x + 2112.43	0.9975	50–2000	3.963	137	1.524
vanillic acid	y = 17.86x − 166.86	0.9973	50–2000	4.865	167	3.196
caffeic acid	y = 1377.37x + 6941.68	0.9984	50–2000	5.046	179	0.041
ferulic acid	y = 108.21x − 1692.97	0.9989	50–2000	10.924	193.1	0.248
cinnamic acid	y = 12.75x − 100.06	0.9983	50–2000	18.060	146.9	0.559
rutin	y = 1017.39x − 35010.60	0.9993	50–2000	11.689	609.1	0.388
quercetin-3-O-glucoside	y = 2782.17x − 74249.70	0.9993	50–2000	11.906	462.8	0.300
kaempferol-3-O-glucoside	y = 2350.39x + 111703.32	0.9952	50–2000	13.776	446.8	ND
myricetin	y = 0.91x − 77.39	0.9953	50–2000	15.033	317	1.021
morin	y = 611.76x + 3378.56	0.9962	50–2000	16.578	301	ND
luteolin	y = 1103.83x + 98837.97	0.9961	50–2000	18.06	285	ND
quercetin	y = 3032.78x − 1438777.72	0.998	100–2000	18.153	301	5.342
kaempferol	y = 10.83x − 72.24	0.9987	50–2000	20.439	271.1	0.126
naringenin	y = 14.58x + 410.22	0.9969	50–2000	20.439	271.1	0.072
pinobanksin	y = 71.63x + 450.56	0.995	50–2000	20.709	271.1	0.384
diosmetin	y = 4225.03x + 392274.94	0.9916	50–2000	21.102	298.9	ND
chrysin	y = 498.99x + 27824.49	0.9989	50–2000	25.205	253.1	ND
pinocembrin	y = 340.00x + 29581.47	0.9902	50–2000	25.739	255.1	ND
galanin	y = 120.24x + 9565.86	0.9952	50–2000	25.907	269	ND
caffeic acid phenethyl ester	y = 15593.82x + 3956352.11	0.9976	100–2000	26.155	283.1	ND

Note: ND means not detected.

**Table 5:** In vitro ECH (extract from *Carthamus tinctorius* L. honey) free radical scavenging activity.

	IC_50_ (µg/mL)
DPPH	68.23 ± 0.40
ABTS+	81.88 ± 0.54

## Supplementary Materials

The following are available online at https://www.mdpi.com/2304-8158/9/8/1039/s1. **Figure S1:** Micrograph of safflower pollen from a safflower honey sample.
