# Supplementary material for: Antioxidant and Anti-Inflammatory Activities of Safflower (Carthamus tinctorius L.) Honey Extract
Source: Foods. 2020 Aug 2;9(8):1039. doi: 10.3390/foods9081039 (PMC7466186; doi:10.3390/foods9081039)
Supplement: Supplementary file 1 [file foods-09-01039-s001.pdf]

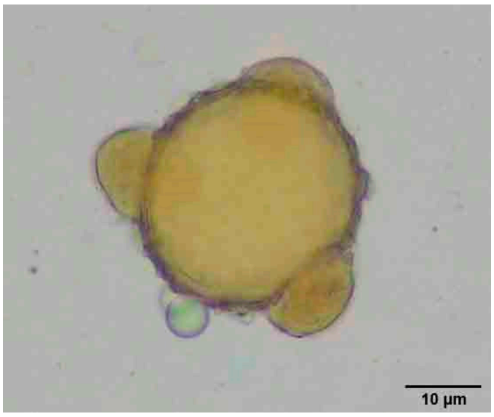

Figure S1. Micrograph of safflower pollen from a safflower honey sample.

In order to determine the plant origin of honey samples from safflower, the pollen in the honey samples was examined microscopically and the results are shown in Figure S1.
